# Research on the Dynamic Hysteresis Loop Model of the Residence Times Difference (RTD)-Fluxgate

**DOI:** 10.3390/s130911539

**Published:** 2013-09-02

**Authors:** Yanzhang Wang, Shujun Wu, Zhijian Zhou, Defu Cheng, Na Pang, Yunxia Wan

**Affiliations:** 1 College of Instrumentation & Electrical Engineering, Jilin University, No.938 Ximinzhu Street, Changchun 130026, China; E-Mails: yanzhang@jlu.edu.cn (Y.W.); zhouzhijian@jlu.edu.cn (Z.Z.); chengdefu@jlu.edu.cn (D.C.); pangna12@mails.jlu.edu.cn (N.P.); wanyx@jlu.edu.cn (Y.W.); 2 Key Laboratory of Geo-Exploration Instrumentation, Ministry of Education, Jilin University, No.938 Ximinzhu Street, Changchun 130026, China

**Keywords:** RTD-fluxgate core, hysteresis loop, arctangent model, simulation of the output response

## Abstract

Based on the core hysteresis features, the RTD-fluxgate core, while working, is repeatedly saturated with excitation field. When the fluxgate simulates, the accurate characteristic model of the core may provide a precise simulation result. As the shape of the ideal hysteresis loop model is fixed, it cannot accurately reflect the actual dynamic changing rules of the hysteresis loop. In order to improve the fluxgate simulation accuracy, a dynamic hysteresis loop model containing the parameters which have actual physical meanings is proposed based on the changing rule of the permeability parameter when the fluxgate is working. Compared with the ideal hysteresis loop model, this model has considered the dynamic features of the hysteresis loop, which makes the simulation results closer to the actual output. In addition, other hysteresis loops of different magnetic materials can be explained utilizing the described model for an example of amorphous magnetic material in this manuscript. The model has been validated by the output response comparison between experiment results and fitting results using the model.

## 1. Introduction

The fluxgate sensor has been widely used in magnetic field measurements due to its high sensitivity, small size and low power consumption [1–3]. The traditional fluxgate sensor employs the output second harmonic to detect the magnetic field, and it has developed slowly because of the probe noise, the production process and the material constraints [4–7]. The RTD-fluxgate sensor developed by Andò *et al.* [8–12], can detect the magnetic field by means of the corresponding relationship between the residence time difference of the output pulse signal and the measured magnetic field. Owing to the outstanding features of the RTD-fluxgate sensor, such as high sensitivity, convenient processing, easy miniaturization and digitization, *etc.*, it has attracted more attention.

The shape of the hysteresis loop which relates to the features of magnetic core determines the final output characteristics of the RTD-fluxgate. Therefore, the precise fitting of the hysteresis loop determines the fluxgate simulation quality [13–16]. Now there are some methods using simple mathematical models that are equivalent to the hysteresis loop, such as the sub-function model proposed by Primdahl *et al.* [17], the polynomial model described by Bornhofft *et al.* [18] and the arc tangent model presented by Trujillo *et al.* [19]. To obtain the changing rule of the hysteresis loop, these methods approximately describe the hysteresis loop by a simple mathematical model. Another category of methods to obtain the changing rule of the hysteresis loop is experimental measurement, and the corresponding numerical relationship is then created based on the measured results [20]. The calibration methods that are usually used in experiments include the oscilloscope method, Hall effect method and electronic integrator method. The oscilloscope method can be simple, intuitive and easily understood, but the measurement parameters are not accurate enough, and can only be used for experimental illustration. The Hall effect and electronic integrator methods are both measured point by point, and they are relatively more accurate, but what they measure are static hysteresis loops which are inconsistent with the dynamic working state of the core when fluxgate is repeatedly magnetized [20–24]. Recently, a behavioral model for the RTD-fluxgate simulation of nonlinear hysteretic device was proposed [25]. The dynamic behavior in the literature [25] is based on a bistable potential energy function [10,26,27]. Additional, the illustrated model parameters, without any physical meaning, are estimated to approximate the real values in a complex procedure. However, the aforementioned model, based on a hysteresis state assume of FeSiB amorphous ferromagnetic core material, is instantaneous during the transition of magnetization process between the two stable states. Although the accuracy of the output signal in time domain is improved as presented in the literature [27], the model cannot reflect the magnetic features of common core materials because of the requirement for the magnetic core hysteresis loop to have a large rectangle ratio.

In order to obtain the accurate fitting of the dynamic changing rule of the hysteresis loop when the RTD-fluxgate is working and facilitate the numerical simulation of this kind of fluxgate, this paper proposes a new arc tangent model containing a dynamic permeability parameter via analysis of the working principle of RTD-fluxgate, ideal hysteresis loop model, and arc tangent model. Compared with the output response results of the RTD-fluxgate based on an ideal hysteresis loop model, the novel arc tangent model which contains a dynamic permeability parameter and fits the actual dynamic hysteresis loop improves the accuracy of the hysteresis loop simulation on soft magnetic materials and reduces the deviation of the output response simulation of the RTD-fluxgate sensor.

## 2. Working Principle of RTD-Fluxgate Sensor

**Figure 1.**
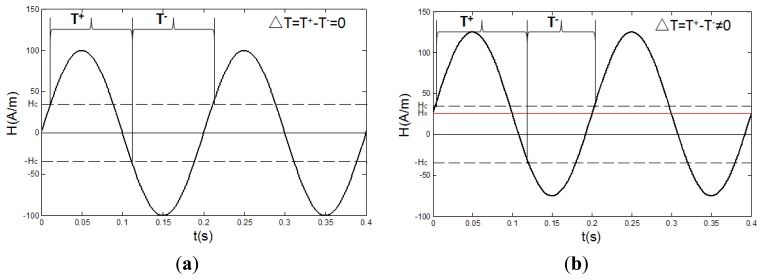
The schematic diagram of the output pulse signals: (**a**) there is no target magnetic field; (**b**) there is a target magnetic field.

When the RTD-fluxgate is working, the core of the sensor is magnetized by a periodically alternating magnetic field to the states of two-way over-saturation. The target magnetic field can influence the residence time of the magnetic core in positive and negative saturation states. In practice, we may obtain the values of target magnetic fields by detecting the time difference of the output pulse signals which relate to the states. If the target magnetic field is zero, since the exciting magnetic field only exists in the axial direction of the sensor, the residence times of the magnetic core in positive and negative saturation states are the same, and the time difference between them is zero, ΔT = T^+^ − T^−^ = 0, and their sum is the excitation signal cycle, T^+^ + T^−^ = T, as is shown in Figure 1a. If a target magnetic field H_x_ exists along the axis of the sensor, this field is superimposed on the excitation magnetic field, so that the residence times of the magnetic core in positive and negative saturation states are different, then the time difference between them is not zero, ΔT = T^+^ − T^−^ ≠ 0, as is shown in Figure 1b [28].

In Figure 1, H_c_ is the coercive field, H_x_ is the target magnetic field, T^+^ is the time interval between the positive pulse and negative pulse of the output signal, and T^−^ is the time interval between the negative pulse and positive pulse of the output signal.

## 3. Foundation of the Fixed Hysteresis Loop Model

As described in the working principle of the RTD-fluxgate, the output response of the sensor is related to the two-way over-saturation and the target magnetic field is detected based on the difference between the residence times in two states. The dynamic hysteresis loop reflects the dynamic working process of the core and the states of output signal, but the magnetization process transition between the two stable states is not instantaneous, therefore, an accurate description of the hysteresis loop can affect the RTD-fluxgate research.

### 3.1. The Relationship between the Dynamic Permeability Parameter and the Output Response of the RTD-Fluxgate Sensor

The features of the flux density *B* are reflected by the coil voltage. Assuming that the excitation signal is a sine wave, as shown in Equation (1):

(1) $$H={\hat{H}}_{e}\sin(\omega t+\theta )$$

The expression of the output signal is shown in Equation (2) below:

(2) $$ɛ=N\cdot \frac{d\phi }{dt}=N\cdot \frac{dB\cdot A}{dt}=N\cdot A\cdot \frac{dB}{dH}\cdot \frac{dH}{dt}=N\cdot A\cdot \frac{dB}{dH}\cdot \omega {\hat{H}}_{e}\cos(\omega t+\theta )$$

According to the Faraday law of electromagnetic induction, under the condition that the sensing component parameters of the sensor and the amplitude of the excitation field are constant, the maximum dynamic permeability of the core determines the maximum amplitude of the output signal.

**Figure 2.**
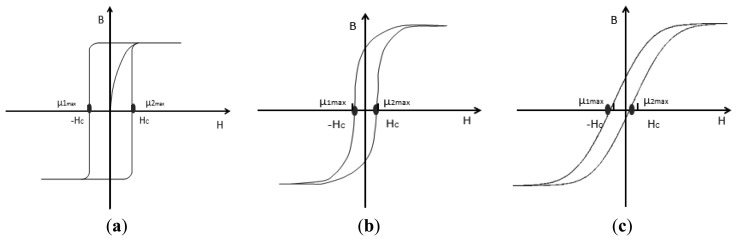
(**a**) The ideal hysteresis loop model. (**b**) The static hysteresis loop model. (**c**) The actual dynamic hysteresis loop model.

The ideal hysteresis loop is shown in Figure 2a, where the curve form is fixed, and the magnetic permeability is infinite at the positions of the coercive field. The static hysteresis loop is shown in Figure 2b, where the curve variation corresponds to the static features of the core, not the actual dynamic changing rule. When the fluxgate is working, the excitation magnetic field not only has to overcome the coercive field H_c_ of core in this direction but also needs to overcome the external magnetic field. That means the coercive field H_c_ is increased in this direction, and *vice versa*. If there is a measured magnetic field, the left and the right branches of the hysteresis loop are asymmetrical, so the maximum voltage amplitude of the output signal will become smaller, as shown in Figure 2c.

**Figure 3.**
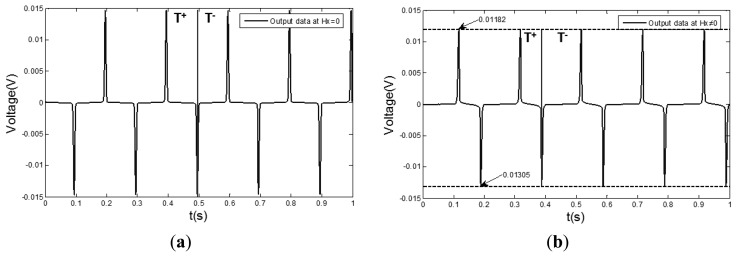
The output signal of the sensor: (**a**) there is an external magnetic field; (**b**) there is no external magnetic field.

In Figure 2, μ_1max_ and μ_2max_ represent the position of the maximum dynamic permeability when the core is reversely and forwardly magnetized, respectively. In order to verify the relationship between the hysteresis loop and the output signal, the experiments are implemented under two different conditions (there is the external magnetic field and there is no external magnetic field), as shown in Figure 3.

As shown in the comparison between Figure 3, if there is a external magnetic field, the amplitude of the output signal becomes smaller and the values of the positive and negative peaks become different. That means that the maximum dynamic permeabilities of the hysteresis loop become smaller simultaneously both in reverse and forward magnetization curves, |μ_1max_| ≠ |μ_2max_|. Figure 3b shows that if the distance between an adjacent positive peak and negative peak is changing, a time difference will emerge. In Figure 3b, the first cycle (0∼0.2 s) of the output signal is taken as an example. The peak appears at the position of maximum permeability of the forward magnetization and the bottom appears at the position of maximum permeability of the reverse magnetization. As the time distance between them becomes smaller, so the positions of μ_1max_ and μ_2max_ are moved and the greater the measured magnetic field is, the more distance the two positions will move toward the same orientation, then the time differences of the output signal will become greater, as shown in Figure 2c. If the direction of the measured magnetic field is reversed, the two positions will move to the opposite direction.

In summary, the caculation of the level of variation of the maximum dynamic permeability between the forward magnetization curve and the reverse magnetization curve in a hysteresis loop can caculate the time difference of the sensor output signal. Because the forward magnetization time and the reverse magnetization time compose a magnetization cycle, the same as the cycle of the magnetic excitation field. The variation of the time difference of the output signal is reflected by the changing of the maximum permeability positions. Therefore, the fitting accuracy of the hysteresis loop, especially at the positions of the maximum permeability, directly affects the output response simulation quality of the RTD-fluxgate established by the fitting equation.

### 3.2. The Dynamic Permeability Model

To improve the fluxgate simulation accuracy, Trujillo *et al.* focused on the output response of the fluxgate through the SPICE simulation mode. Based on the shape features of the hysteresis loop, the arc tangent model is established via its trigonometric function, as shown in following equation [19]:

(3) $$B(H)=(2B_{\text{sat}}/\pi )\arctan(H/H_{0})$$

where:

(4) $$H_{0}=2B_{\text{sat}}/\pi \mu_{0}\mu_{d}$$

In Equations (3) and (4), *B* is the core flux density, B_sat_ is the saturation flux density, μ_0_ is the permeability in vacuum state, *H* is excitation magnetic field and μ_d_ is the value of relative permeability of core when *H* = 0.

Based on Equation (3), the saturation flux density parameter α replaces 2*B_sat_*/*π* and the permeability parameter β replaces 1/H_0_. In order to improve the fitting accuracy of the hysteresis loop model, taking into account that the coercive field in the hysteresis loop is always hindering the relative change of the excitation magnetic field, the correction term ±H_c_ is included in the arc tangent model, so Equation (3) can be revised as Equation (5):

(5) B(H)=α⋅arctan[β⋅(H±Hc)]

In the model described by Equation (5), the permeability parameter *β* is a pending constant. Through appropriate transformation of Equation (5), Equation (6) can be given by:

(6) $$\beta =\tan(B/\alpha )/(H\pm H_{c})$$

According to Faraday's law of electromagnetic induction, the core flux density *B* is the integral of output signal amplitude, so the core flux density can be obtained by measuring the induced voltage of the RTD-fluxgate second coil, and then under the condition that α, *H* and H_c_ are known, the values of the permeability parameter *β* can be calculated using Equation (6).

**Figure 4.**
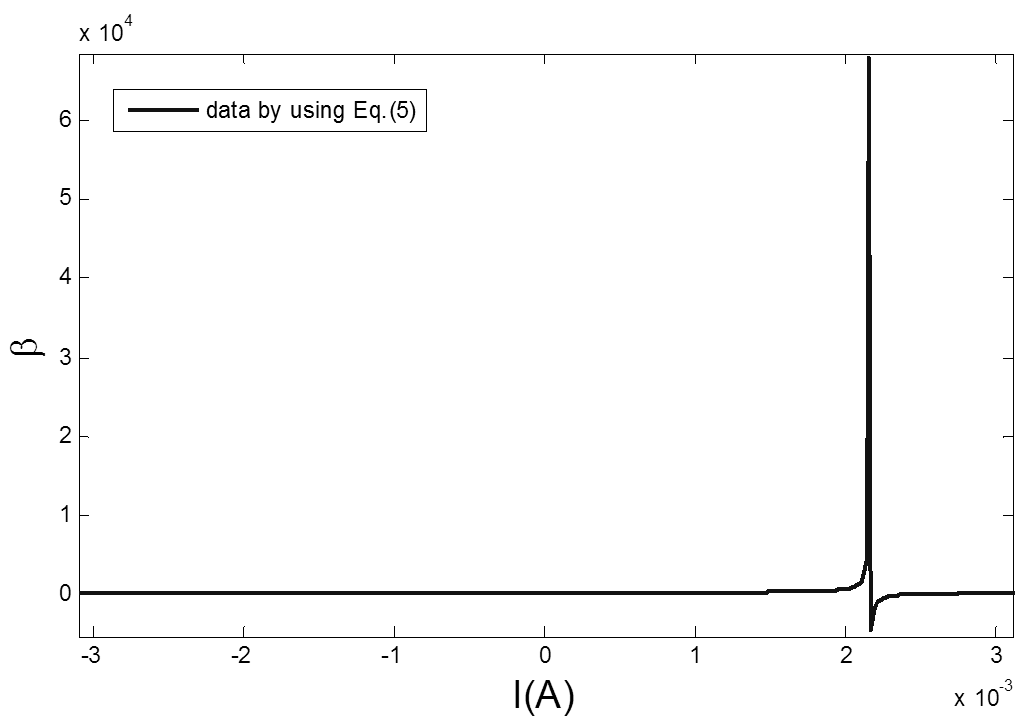
The actual changing curve of the permeability parameter *β*.

In a magnetically shielded room, a precision current source (KEITHLEY 6221) is used to drive a Helmholtz coil to generate a fixed external magnetic field. A high precision data acquisition module (NI PXI-4495) is used to collect the sensor output signals under the condition of a 100 mA, 5 Hz sine excitation field. The measured induction voltage is integrated to get the changing rules of the core flux density *B*. The section permeability parameter *β* of the curve is obtained by taking the reverse magnetization data *B* of the hysteresis loop into Equation (6), as shown in Figure 4. The permeability parameter *β* is varied by the changing of the excitation magnetic field *H*.

Figure 4 shows that the arc tangent model to which only the correction term of coercive field is added may not accurately reflect the dynamic permeability parameter *β* of the hysteresis loop. Taking into account that the changing characteristics of the *β* curve fits the linear characteristic of the Lorentz function, *i.e.*, the permeability parameter increases sharply when the amplitude of the excitation magnetic field is close to the coercive field, and *vice versa*. Through matching the high order fitting terms based on the Lorentz function, the permeability parameter *β* in Equation (5) is replaced as the dynamic permeability parameter *β_D_*, as shown in Equation (7):

(7) $$\beta_{D}(H)=\overset{N}{\underset{n=1}{\text{∑}}}P_{n}\cdot {\left(H\pm Hc\right)}^{n-2}$$

In Equation (7), *P_n_* is a fitting parameter which can be obtained by fitting the actual permeability parameters from Equations (5) and (6). The value of N can be appropriately selected according to the precision requirement. The arc tangent model that contains the dynamic permeability parameter is shown in Equation (8):

(8) $$B(H)=\alpha \cdot \arctan[\beta_{D}\cdot (H\pm Hc)]$$

where the parameter *β_D_* is adjusted by considering the characteristics of the core relative permeability and the effects of the external magnetic field, so the μ_d_ in Equation (4) is a variable value.

**Figure 5.**
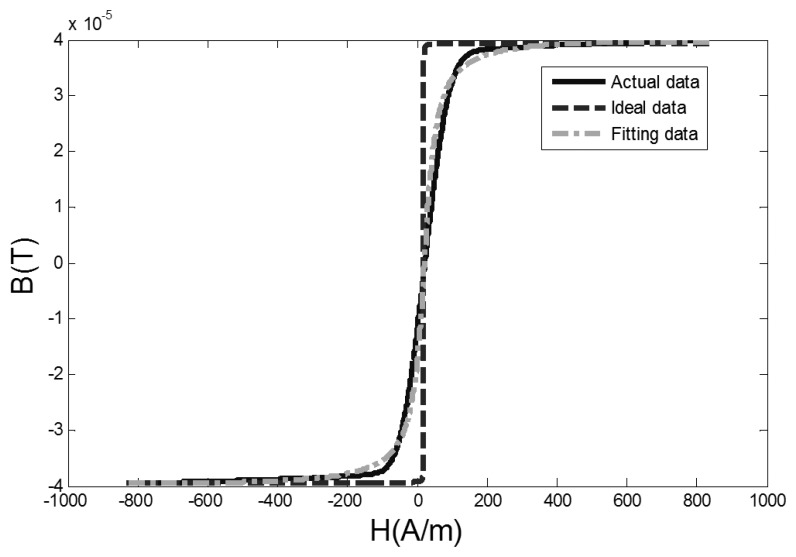
The comparison between actual measuring curve and two fitting curves of the hysteresis loop.

By using the Matlab software, when the excitation magnetic field *H* is known and the core flux density *B* is obtained by taking integration with the output signal amplitude, the measured data of hysteresis loop are fitted by Equation (8) with an external magnetic field of 0.4 A/m. The comparison among actual data, fitted data and the ideal hysteresis loop is shown in Figure 5.

**Figure 6.**
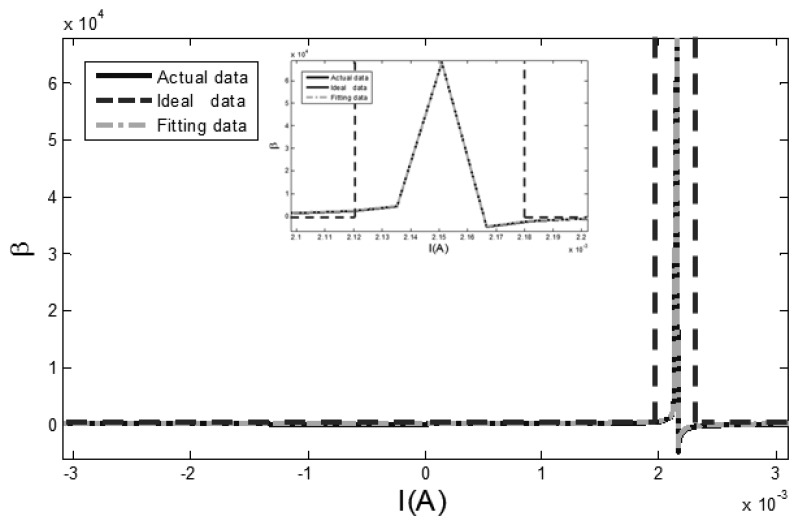
The contrast curve of dynamic permeability parameters.

In Figure 5, the actual data is the measured hysteresis loop, the ideal data is the ideal hysteresis loop, and fitting data is the hysteresis loop fitted by Equation (8). The difference of dynamic permeability parameters between actual data, ideal data and fitting data curves is shown in Figure 6.

In Figure 6, the relatively flat parts reflect the changing dynamic permeability parameter of the hysteresis loop, which is near the position of the saturatured flux density. The varying parts in the middle of Figure 6 demonstrate the sharp changing of the dynamic permeability parameter in the intermediate part of the hysteresis loop, and its peak point is the maximum permeability, which is close to the coercive field. This point is the peak of the RTD-fluxgate sensor output signal in accordance with Faraday's law of electromagnetic induction.

As seen from Figure 6, there is a significant difference between the ideal hysteresis loop and the actual hysteresis loop concerning the dynamic permeability parameter, because the dynamic permeability parameter *β* of the ideal hysteresis loop is an infinite value. Meanwhile, the arc tangent model (8), containing a dynamic permeability parameter, makes up the lack of the ideal hysteresis loop and adjusts the permeability parameter *β* appropriately and makes the curvature of hysteresis loop model (*i.e.*, the core permeability) correct dynamically with the changing of the excitation magnetic field. The proposed model describes the features of the core hysteresis loop more accurately when the RTD-fluxgate is working. Therefore the features of the core materials, such as permeability μ, saturation flux density B_sat_ and coercive field H_c_ can be reflected intuitively by the BH curve. This is beneficial for the selection of core materials. The fitting data of the dynamic permeability parameter *β_D_* fitted by the Equation (7) is similar to the actual data of the dynamic permeability parameter in Figure 6.

**Figure 7.**
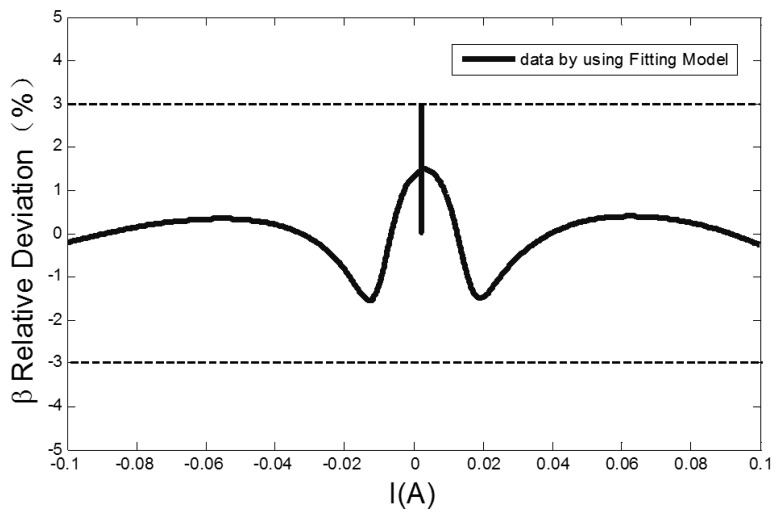
The relative deviation between the fitted dynamic permeability parameter and the actual dynamic permeability parameter.

Because the output signal peaks of RTD-fluxgate sensor correspond to the positions of maximum core dynamic permeability, and the fitting of dynamic permeability parameter affects the output response simulation of the RTD-fluxgate. As seen in Figure 7, the relative deviation of the dynamic permeability parameter fitted by the Equation (7) is less than ±3%, which is useful for researching the output response of RTD-fluxgate sensor.

## 4. Analysis of the RTD-Fluxgate Output Response Based on the New Model

### 4.1. The RTD Fluxgate Output Response Based on the Ideal Hysteresis Loop

In the ideal condition that the maximum permeability of hysteresis loop at the position of coercive field is infinite, Andò *et al.* proposed the output response of RTD-fluxgate under a sine excited magnetic field, as shown in Equation (9) [29–31]:

(9) $$\Delta T_{\sin}=\frac{2}{w}[\arcsin(\frac{H_{c}+H_{x}^{\mathrm{int}}}{{\hat{H}}_{e}})-\arcsin(\frac{H_{c}-H_{x}^{\mathrm{int}}}{{\hat{H}}_{e}})]$$

In Equation (9), *Ĥ_e_* is the maximum magnetic field of excitation signal, $H_{\text{x}}^{\mathrm{int}}$ is the magnetic field inside the core; ω is the angular frequency of the excitation signal, H_c_ is the coercive field. As the output response of the RTD fluxgate is established based on the ideal hysteresis loop model and the ideal excitation signal, the relative permeability of the actual hysteresis loop is not an infinite value which is changed while the vary of excitation magnetic field. Therefore, as the positions of the maximum permeability cannot be confirmed accurately from the time difference output response of the RTD-fluxgate which is based on the ideal hysteresis loop model. There is a time difference deviation owing to the fact the hysteresis state transition time of the core status can be neglected.

### 4.2. The RTD-Fluxgate Output Response Based on the Dynamic Hysteresis Loop

In order to verify the accuracy of the proposed model and analyze the output response of the RTD-fluxgate conveniently, another type of analysis of the output response of the RTD-fluxgate is obtained by using Equation (8). According to the RTD-fluxgate output principle, the fluxgate output signal is the derivative of the core magnetic induction, so the output induction voltage signal of the fluxgate sensor in the corresponding excitation magnetic field can be obtained by deriving the magnetic induction of the fitting signal, as shown in Equation (10):

(10) $$ɛ=\frac{dB}{dt}=\alpha \cdot \frac{dH}{dt}\cdot \frac{\overset{N}{\underset{n=1}{\text{∑}}}\left[P_{n}\cdot (n-1)\cdot {\left(H\pm Hc\right)}^{n-2}\right]}{1+{\beta_{D}}^{2}\cdot {\left(H\pm Hc\right)}^{2}}$$

The output response of the RTD-fluxgate is proportional to the magnetic field of the measured target, so the time difference can be obtained through the time point related to the output signal peaks by derivating Equation (10). After the deriviation, the form of Equation (10) is changed as shown in Equation (11):

(11) $${ɛ}^{\prime }=\frac{\alpha .\left\{\frac{d^{2}H}{dt^{2}}\cdot \overset{N}{\underset{n=1}{\text{∑}}}[P_{n}\cdot (n-1)\cdot {\left(H\pm Hc\right)}^{n-2}]+{\left(\frac{dH}{dt}\right)}^{2}\cdot \overset{N}{\underset{n=1}{\text{∑}}}\left[P_{n}\cdot (n-1)\cdot (n-2)\cdot {\left(H\pm Hc\right)}^{n-3}\right]\right\}}{1+{\beta_{D}}^{2}{\left(H\pm Hc\right)}^{2}}-\frac{2\alpha \cdot \beta_{D}\cdot \frac{dH}{dt}\cdot \overset{N}{\underset{n-1}{\text{∑}}}\left[P_{n}\cdot (n-1)\cdot {\left(H\pm Hc\right)}^{n-2}\right]\cdot \left\{\overset{N}{\underset{n=1}{\text{∑}}}\left[P_{n}\cdot (n-2)\cdot {\left(H\pm Hc\right)}^{n-1}\right]+\beta_{D}\cdot (H\pm HC)\cdot \frac{dH}{dt}\right\}}{{\left[1+{\beta_{D}}^{2}{\left(H\pm Hc\right)}^{2}\right]}^{2}}$$

**Figure 8.**
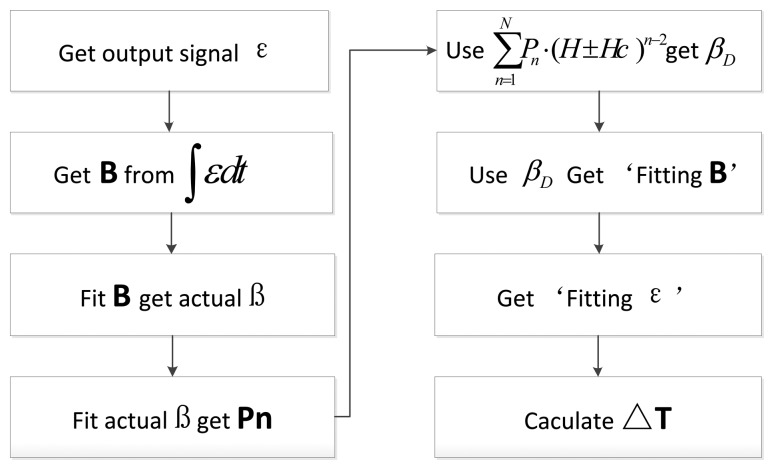
The flow chart of the output response of the RTD-fluxgate by the fitting model.

There exists only a numerical result, not an analytical result because Equation (11) is the transcendental equation. In order to verify the output response effectis, which are based on the time difference of the dynamic hysteresis loop, the following treatments are done and the details of the process are shown in Figure 8.

As shown in Figure 8, firstly, the output voltage signal of the sensor can be obtained through experiments, and integrated to get actual flux density *B* that plays an action on the core. Under the condition that α, *H* and H_c_ are known, the value of the actual dynamic permeability parameter *β* can be calculated according to Equation (6). The fitting parameter P_n_ can be calculated through Equation (7). Then an arc tangent model based on the dynamic permeability parameter and fitting core flux density *B* can be obtained by the combination of Equation (8) and *β_D_* which is derived from Equation (7). Finally, the time difference output response of the RTD-fluxgate under the corresponding conditions can be calculated by Equations (10) and (11).

**Figure 9.**
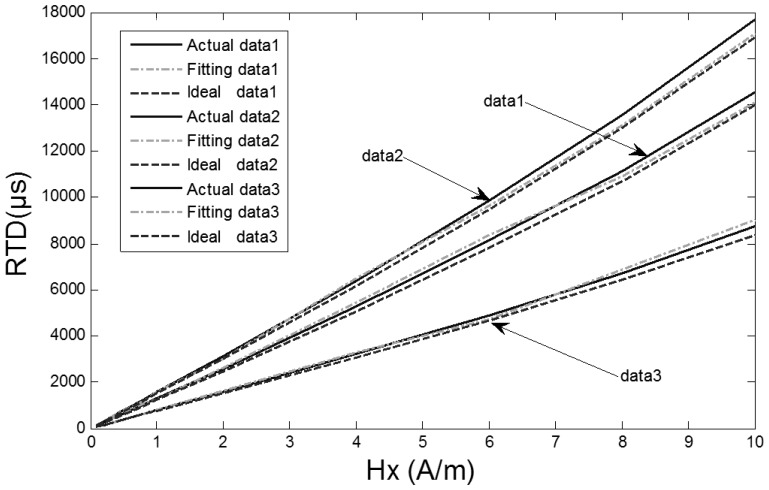
The output time D-values contrast of the RTD-fluxgate. Data1 is the output time D-value in the (100 mA, 5 Hz) sine excitation magnetic field. Data2 is the output time D-value in the (80 mA, 5 Hz) sine excitation magnetic field. Data3 is the output time D-value in the (100 mA, 10 Hz) sine excitation magnetic field.

The experiments are validated under the conditions of excitation magnetic fields 100 mA sine at 5 Hz, 80 mA sine at 5 Hz and 100 mA sine at 10 Hz and a range of external magnetic fields from 0.08 A/m to 10 A/m with a 2.0 A/m interval in the magnetically shielded room. A couple of output signals are selected for fitting. The output time D-values of the RTD-fluxgate which are actually measured, and those calculated by Equations (9) and (11) are shown in Figure 9, respectively.

**Figure 10.**
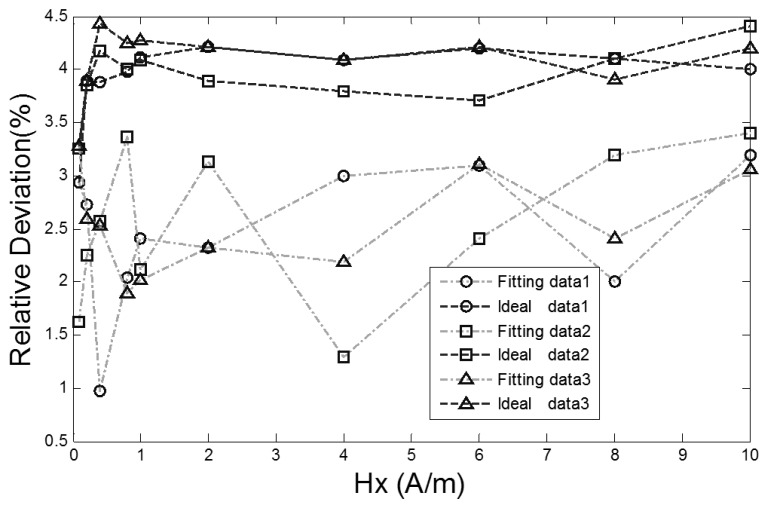
The relative deviation contrast curves between the output time D-values of the RTD-fluxgate under two different conditions s and the actual value.

As seen from Figure 9, the output time D-values of the RTD-fluxgate calculated by Equation (11) approximate the output time D-values which are actually measured. The relative deviation contrast curves between the D-values of the RTD fluxgate output time under two different conditions (Equation (9) or (11)) and the actual values are shown in Figure 10.

The corresponding relative deviation contrast values are 2.9%–4.4% and 1.0%–3.4%, respectively. The sensitivity of the proposed RTD-fluxgate output response is closer to the practical application compared with the math model, so the proposed model can minimize the deviation between the magnetic features of the core material in simulation and the practical application materials.

## 5. Conclusions

A special arc tangent model is proposed to make the relative fitting deviation of the hysteresis loop less than ±3%. The described model changes the original permeability parameter to the dynamic permeability parameter and involves the coercive field in the excitation magnetic field. The physical parameters α, *β_D_* and H_c_ can describe the hysteresis loop more accurately. The model is useful for research on the output response of RTD-fluxgate sensors and selecting the core materials. The absolute values of relative deviation between the output time D-values of the RTD fluxgate and the actual values are less than 3.4%. In addition, the illustrated model makes up for the drawback that the fixing shape of the ideal hysteresis loop model could not accurately reflect the actual dynamic variation of the hysteresis loop, fits the core hysteresis loop more accurately, and minimizes the deviation between the magnetic features of the core material in simulation and practical application materials. The model provides a theoretical basis for research on the simulation of the sensors' output responses.
